# Statin pre-treatment is associated with lower platelet activity and favorable outcome in patients with acute non-cardio-embolic ischemic stroke

**DOI:** 10.1186/cc10303

**Published:** 2011-07-08

**Authors:** Nai-Wen Tsai, Tsu-Kung Lin, Wen-Neng Chang, Chung-Ren Jan, Chi-Ren Huang, Shang-Der Chen, Kuei-Yueh Cheng, Yi-Fang Chiang, Hung-Chen Wang, Tzu-Ming Yang, Yu-Jun Lin, Wei-Che Lin, Hsueh-Wen Chang, Lian-Hui Lee, Cheng-Hsien Lu

**Affiliations:** 1 Departments of Neurology, Kaohsiung Chang Gung Memorial Hospital, Chang Gung University College of Medicine, 123 Ta Pei Road, Niao Sung Hsiang, Kaohsiung, Taiwan; 2 Department of Medical Education and Research, Kaohsiung Veterans General Hospital, 386 Ta-Chung 1st RD, Kaohsiung, 81346, Taiwan; 3 Departments of Nursing, Kaohsiung Chang Gung Memorial Hospital, Chang Gung University College of Medicine, 123 Ta Pei Road, Niao Sung Hsiang, Kaohsiung, Taiwan; 4 Departments of Research, Kaohsiung Chang Gung Memorial Hospital, Chang Gung University College of Medicine, 123 Ta Pei Road, Niao Sung Hsiang, Kaohsiung, Taiwan; 5 Departments of Neurosurgery, Kaohsiung Chang Gung Memorial Hospital, Chang Gung University College of Medicine, 123 Ta Pei Road, Niao Sung Hsiang, Kaohsiung, Taiwan; 6 Departments of Radiology, Kaohsiung Chang Gung Memorial Hospital, Chang Gung University College of Medicine, 123 Ta Pei Road, Niao Sung Hsiang, Kaohsiung, Taiwan; 7 Department of Biological Science, National Sun Yat-Sen University, 70 Lien-Hai Road, Kaohsiung, Taiwan

**Keywords:** flow cytometry, ischemic stroke, outcome, platelet activation

## Abstract

**Introduction:**

Statins reportedly have anti-inflammatory and anti-thrombotic effects aside from cholesterol-lowering. This study aimed to evaluate the effect of pre-existing statin use on platelet activation markers and clinical outcome in acute ischemic stroke patients.

**Methods:**

This prospective study evaluated 172 patients with acute ischemic stroke divided in two groups: patients with pre-existing statin (*n *= 43) and without pre-existing statin (66 cases with statins initiated post-stroke and 63 without statin treatment). Platelet activation markers (CD62P and CD63) were measured by flow cytometry at different time points after stroke and analyzed with clinical outcome.

**Results:**

The CD62P and CD63 expressions on platelets were significantly lower in the patients with pre-existing statin use compared to the patients without pre-existing statin use on Day 1 post-stroke (*p *< 0.05). The CD62P expression was significantly lower in the patients with pre-existing statin use on 90 days after the acute stroke (*p *< 0.05). Patients with pre-existing statin use had lower incidences of early neurologic deterioration (END) than those without treatment (*p *< 0.05). Among several baseline clinical variables, admission NIHSS score, history of coronary artery disease, and pre-existing statin use were independent predictions of good clinical outcome at three months.

**Conclusions:**

Pre-existing statin use is associated with decreased platelet activity as well as improved clinical outcome and reduced END in patients with acute ischemic stroke.

## Introduction

Stroke is a major cause of morbidity and one of the leading causes of death worldwide [1]. Atherothrombosis and inflammation play important roles in the pathogenesis of acute ischemic stroke [2-4]. A previous study demonstrates that platelet activity, measured by CD62P and CD63 expressions on platelets, are increased after acute ischemic stroke and reduced in patients who receive anti-platelet therapy [5-7]. Anti-platelet drugs are the most commonly used drugs for secondary prevention after ischemic stroke of non-cardio-embolic origin [8], but their efficacy is not completely satisfactory [9,10].

Statins, the 3-hydroxy 3-methyl-glutaryl coenzyme-A (HMG-CoA) reductase inhibitors, are medications originally used for the control of hypercholesterolemia [11]. However, there is increasing evidence that statins have anti-inflammatory and anti-thrombotic effects aside from their cholesterol-lowering effect [12,13]. Statin therapy has been shown to reduce cardiovascular events, including myocardial infarction, stroke, and death [14-16]. Moreover, early statin treatment may reduce the severity and improve the outcome of myocardial infarction, ischemic stroke, and intra-cerebral hemorrhage [17-20].

Although statin therapy is widely used in patients at high-risk for major vascular events, the benefits of pre-existing statin therapy in patients with acute ischemic stroke remain controversial. Multiple studies have demonstrated improved clinical outcomes in patients taking statins at stroke onset [18,19]; however, mechanisms conferring this protection have not been well studied. Thus, this prospective cohort study aimed to test the difference in platelet activity between patients taking statins before and after acute ischemic stroke by assessing CD62P and CD63 expression. This study also analyzed if prior statin treatment could reduce the neurologic deterioration and improve the functional outcome of patients with ischemic stroke.

## Materials and methods

### Study participants

Consecutive patients with acute ischemic stroke admitted to the Department of Neurology of Chang Gung Memorial Hospital-Kaohsiung, Taiwan, from August 2009 to July 2010 were evaluated. Acute ischemic stroke was defined as acute-onset loss of focal cerebral function persisting for at least 24 hours. The diagnosis of stroke was made based on clinical presentation, neurologic examination, and results of brain magnetic resonance imaging (MRI) with magnetic resonance angiography (MRA). Patients with cardio-embolic stroke were excluded, as well as those with underlying neoplasm, vasculitis, hematologic disorders that affect platelet count or function, end-stage renal disease, liver cirrhosis, and congestive heart failure. As pathogenesis and treatment could be different between patients with cardio-embolic and non-cardio-embolic ischemic stroke, those with cardio-embolism were excluded by clinical presentation, ECG, and cardiac ultrasound, while those who received intravenous thrombolytic therapy were also excluded.

To avoid the confounding factor of anti-platelet therapies or dosage effects on measured platelet activity, all patients taking one or more anti-platelet medication (e.g. aspirin, dipyradimole, or clopidogrel) prior to stroke onset were excluded from enrollment. All enrolled patients were treated with aspirin (100 mg/day) therapy post-stroke. The Institutional Review Committee on Human Research approved the study protocol and the participating subjects provided informed consent.

Demographic data, history of risk factors (i.e., hypertension, diabetes mellitus, dyslipidemia, cigarette smoking, and cardiovascular disease), and history of previous vascular events (i.e., myocardial infarction, angina, old stroke history) were obtained at baseline. Vascular risk factors included hypertension, blood pressure above 140/90 mmHg at two readings or use of anti-hypertensives; diabetes mellitus (DM), elevated blood glucose at two recordings, elevated hemoglobin A1c (HbA1c) or use of anti-diabetics; and dyslipidemia, total cholesterol above 200 mg/dL, triglycerides above 180 mg/dL or use of lipid-lowering medication [10].

Of the 220 patients with acute non-cardio-embolic ischemic stroke, 30 were excluded due to previous anti-platelet therapy before the stroke, six due to cardio-embolic stroke (e.g. paroxysmal atrial fibrillation on EKG), four due to hemorrhagic transformations of ischemic strokes on the first brain imaging, four with end-stage renal disease, and four with gastro-intestinal bleeding in the acute stage. The remaining 172 patients were classified into two groups: patients with pre-existing statin use (patients taking statins prior to stroke onset) and patients without pre-existing statin use (patients did not take statins prior to stroke onset). For further comparison, the patients without pre-existing statin use were divided to two sub-groups: the statin-initiated group (patients placed on statins after stroke onset) and the non-statin treatment group (patients not placed on statins before and after stroke onset).

The lipid-lowering regimens used for preventing ischemic stroke were according to the American Heart Association/American Stroke Association guidelines for diabetic or high-risk patients and included statins if the low density lipoprotein (LDL) was above 70 and in non-diabetics if the LDL was above 100 [21]. The etiologic sub-types of i schemic stroke were classified according to the TOAST (Trial of Org 10172 in Acute Stroke Treatment) criteria [22].

### Clinical assessments

Detailed medical history was obtained from patients and their families whenever possible, with specific standardized questioning regarding prior use of drugs. All of the patients underwent complete neurologic examination upon enrollment and on follow up. Brain MRI with MRA, extra-cranial carotid sonography, and trans-cranial color-coded sonography were performed on ischemic stroke patients during hospitalization. Early neurologic deterioration (END) was defined as an increase of four or more points in National Institutes of Health Stroke Scale (NIHSS) during hospitalization [23]. Follow-up brain CT scans were performed if END was noted, including hemorrhagic transformation (hyperdense signal over the infarction area), enlarged cerebral infarction (increased area of original lower attenuation or new infarction lesions), and cerebral edema (abnormal hypodense signal surrounding the infarction area with sulcal effacement or mass effect) [24].

Neurologic deficits due to stroke were assessed using the NIHSS. Physical disability and handicap were evaluated using the Barthel index (BI) and modified Rankin Scale (mRS). Therapeutic outcomes were evaluated three months post-stroke and good outcome was defined as a three-month mRS of 0 to 2, while poor outcome was mRS of 3 to 6.

### Blood sampling and assessment of platelet activity

Blood samples were collected by venipuncture of the forearm veins in acute stroke patients within 48 hours and on days 7 and 30 post-stroke. Flow cytometry for platelet activity markers was performed as previously described [7]. Platelet activity was assessed using platelet activation markers (CD62P and CD63). Briefly, sodium citrate containing blood was centrifuged for 15 minutes at 1,500 rpm at room temperature. The samples were incubated with saturating concentrations of phycoerythrin (PE)-labeled antibodies (Becton Dickinson Biosciences, CA, USA.) against CD62P (clone AK-4) and CD63 (Clone H5C6) with fluorescein isothiocyanate-labeled antibodies against CD61 (clone VI-PL2) for 30 minutes at room temperature in the dark. For control experiments, platelets were incubated with PE-coupled unspecific mouse IgG1 (Becton Dickinson Biosciences, CA, USA.) with the same ratio and concentration of fluorochrome-to-protein as specific IgG.

After immuno-labeling, the samples were analyzed by Coulter Epics XL flow cytometry (Beckman Coulter, Miami, USA.). Forward light scatter and CD61 expression were used for platelet identification. Platelet-bound anti-CD62P and anti-CD63 antibodies were determined by analyzing 10,000 platelets for PE-positive fluorescence.

Results were presented as percentages of antibody-positive platelets. Intra-assay variability based on repeated measurements of the same blood sample was low, with mean coefficients of variance of 7.5% (< 48 hours), 7.3% (day 7), and 6.9% (day 30) for stroke patients.

### Statistical analysis

Data were presented as mean ± standard error of the mean and as comparisons between groups. Continuous variables, including age, cell counts, lipid profile, HbA1c, blood pressure, and CD62P and CD63 levels were analyzed by independent *t*-test between groups. The NIHSS score between the two groups were analyzed by the Mann-Whitney *U *test. Chi-square test or Fisher's exact test were used to compare proportions between the two patient groups. Repeated measures of ANOVA were used to compare platelet activity at different time points (< 48 hours and on Days 7 and 30 post-stroke), while Scheffe's multiple comparison analyzed the intra-individual course of parameters over time and compared the parameters of two different groups.

The independent *t*-test was used to compare the good and poor outcome groups. Multiple logistic regression analyses were used to determine the independent influence of different predictive variables on clinical outcome. The variables considered were age, gender, lipid profiles, coronary artery diseases, NIHSS score, pre-existing statin use, the levels of CD62P and CD63. A *p *< 0.05 was considered statistically significant. All statistical calculations were performed using the SAS software package, version 9.1 (2002, SAS Statistical Institute, Cary, North Carolina).

### Ethics approval

The study was approved by Chang Gung Memorial Hospital's Institutional Review Committee on Human Research.

## Results

### Baseline characteristics between the patient groups

The group with pre-existing statin use had 43 patients whereas the group without pre-existing statin use had 129. Of these 129 patients, 66 were given statins after the stroke onset (statin-initiated group) and 63 were not given statins at all (non-statin treatment group). In patients with pre-existing statin use, 17 used atorvastatin (10 to 20 mg/d), 11 used fluvastatin (80 mg/d), 12 used rosuvastatin (5 to 10 mg/d), and 3 used simvastatin (10 to 40 mg/d). All of the patients with pre-existing statin use took statins for more than seven days and the final dose of statins was 24 hours before the onset of symptoms. In the statin-initiated group, 18 used atorvastatin (10 to 20 mg/d), 15 used fluvastatin (80 mg/d), 25 used rosuvastatin (5 to 10 mg/d), and 8 used simvastatin (10 to 40 mg/d). They all took the first dose of statin within 72 hours after stroke onset.

The demographic data of the study patients are shown in Table 1. The history of DM was significantly different between the two groups but there were no significant differences in age, sex, other vascular risk factors, and stroke sub-types. The median (inter-quartile range) NIHSS score on admission was significantly lower in patients with pre-existing statin use than those without pre-existing statin use (*p *< 0.05).

**Table 1:** Baseline characteristics of stroke patients in the two groups

	With pre-existing statin use (*n *= 43)	Without pre-existing statin use (*n *= 129)	*P *value
Age (years) (mean ± SEM)	67.5 ± 1.4	64.0 ± 1.2	0.43
Male (%)	69.8	60.6	0.41
Hypertension (%)	83.7	74.2	0.39
Diabetes mellitus (%)	58.1	31.8	0.01
Coronary artery diseases (%)	7.0	3.0	0.37
Intracranial atherosclerosis (%)	30.2	24.2	0.64
Stroke subtype			
Small-vessel disease	51.3	60.0	0.42
Large-vessel disease	48.7	40.0	
Median NIHSS (IQR) scores on admission	4 (2.0-7.0)	5 (2.5-7.75)	0.03

IQR, inter-quartile range; NIHSS, National Institutes of Health Stroke Scale; SEM, standard error of the mean.

Laboratory data were presented in Table 2. Serum total cholesterol, LDL-cholesterol, and triglyceride were significantly lower in patients with pre-existing statin use than those without pre-existing statin use. There were no significant differences between the two groups in terms of white blood cell (WBC), red blood cell (RBC) and platelet counts, HbA1c, prothrombin time (PT), activated partial thromboplastin time (APTT), high density lipoprotein (HDL)-cholesterol, and systolic and diastolic blood pressure. The CD62P and CD63 expressions on platelets were significantly lower in patients with pre-existing statin use than those without pre-existing statin use (*p *< 0.05).

**Table 2:** Baseline laboratory data of stroke patients in the two groups

	With pre-existing statin use (*n *= 43)	Without pre-existing statin use (*n *= 129)	*P *value
White blood cells (X10^3^/ml)	7.5 ± 0.4	7.5 ± 0.3	0.86
Red blood cells (X10^6^/ml)	4.6 ± 0.1	4.7 ± 0.1	0.71
Platelet counts (X10^4^/ml)	20.2 ± 1.1	21.9 ± 0.7	0.43
Total cholesterol (mg/dl)†	148.3 ± 4.3	218.8 ± 6.5	< 0.0001
LDL-cholesterol (mg/dl)†	83.0 ± 3.7	142.9 ± 5.1	< 0.0001
HDL-cholesterol (mg/dl)†	40.3 ± 1.6	42.8 ± 1.5	0.27
Triglyceride (mg/dl)	125.3 ± 8.3	176.3 ± 14.7	0.04
HbA1c (%)	7.3 ± 0.3	7.1 ± 0.3	0.43
APTT (second)	28.0 ± 0.5	27.9 ± 0.4	0.91
PT (second)	10.2 ± 0.1	10.1 ± 0.1	0.37
Systolic BP (mmHg)	138.7 ± 2.4	146.6 ± 2.4	0.13
Diastolic BP (mmHg)	80.1 ± 1.3	85.2 ± 1.6	0.10
CD62P (%)†	2.2 ± 0.6	3.9 ± 0.5	0.01
CD63 (%)†	1.2 ± 0.2	1.8 ± 0.5	0.03

APTT, activated partial thromboplastin time; BP, blood pressure; HbA1c, hemoglobin A1c; HDL, high density lipoprotein; LDL, low density lipoprotein; PT, prothrombin time.

† The data was expressed as primitive data, and the *P *value was obtained from those primitive data that were logarithmically transformed to improved normality and compared by independent -*t *test.

### Serial changes in platelet activation markers between groups

**Figure 1:**
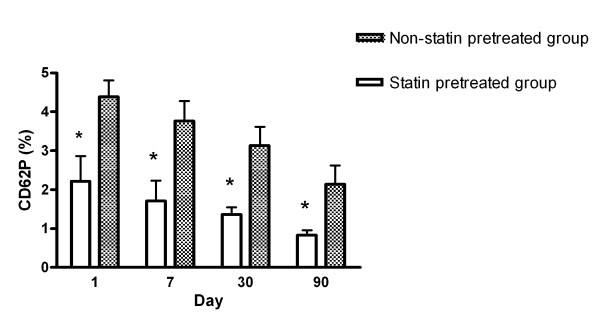
**Serial changes of the percentages of CD62P expression in platelets between patients with pre-existing statin use and those without pre-existing statin use after acute ischemic stroke**. *p < 0.05 patients with pre-existing statin use compared with patients without pre-existing statin use.

Expressions of CD62P between the patients with pre-existing statin use and without pre-existing statin use are shown in Figure 1. The percentage of platelets expressing CD62P was significantly lower in the patients with pre-existing statin used group on day 1 compared with those without pre-existing statin use. Although CD62P levels declined gradually in two groups, platelet CD62P levels were still significantly lower in patients with pre-existing statin use 90 days after the acute stroke (*p *< 0.05). Repeated analysis of variance (ANOVA) with Scheffe's multiple comparison showed that CD62P expressions between the two groups at four different time points (< 48 hours and on days 7, 30, and 90 post-stroke) were significantly different (*p *< 0.05).

**Figure 2:**
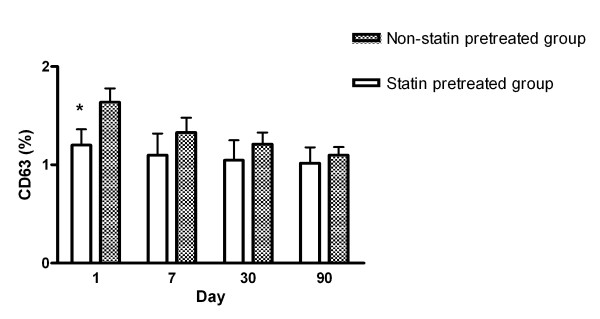
**Serial changes of the percentages of CD63 expression in platelets between patients with pre-existing statin use and those without pre-existing statin use after acute ischemic stroke**. *p < 0.05 patients with pre-existing statin use compared with patients without pre-existing statin use.

Expressions of CD63 between the patients with pre-existing statin use and those without pre-existing statin use are shown in Figure 2. The percentage of platelets expressing CD63 was significantly lower in patients with pre-existing statin use on day 1 than those without pre-existing statin use. However, there was no significant difference after day 7 post-stroke. Repeated measures comparison showed that CD63 expressions between the two groups at different time points were not different.

**Figure 3:**
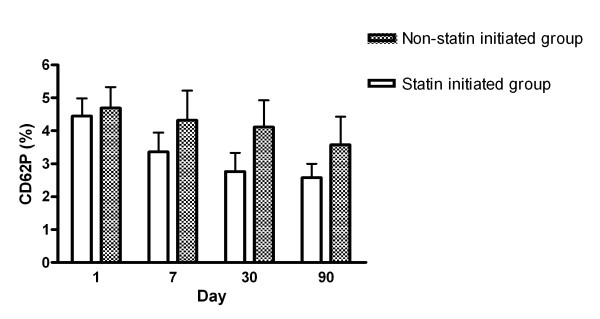
**Serial changes of the percentages of CD62P expression in platelets between statin initiated group and non-statin treated group after acute ischemic stroke**.

**Figure 4:**
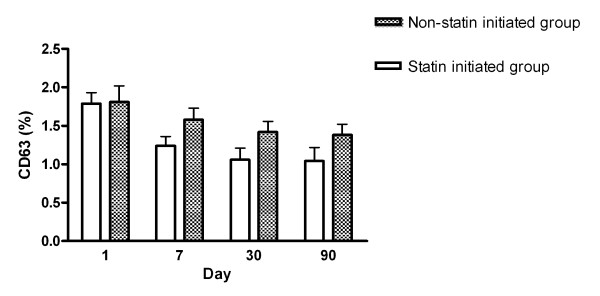
**Serial changes of the percentages of CD63 expression in platelets between statin initiated group and non-statin treated group after acute ischemic stroke**.

Expression of CD62P and CD63 between the statin-initiated and non-statin treatment groups are shown in Figures 3 and 4, respectively. The percentage of platelets expressing CD62P or CD63 was not different between the two sub-groups from days 1 to 90. Repeated measures comparison of CD62P or CD63 expressions at different time points were also not different between the two sub-groups.

### Predictive factors for three-month outcome

Potential prognostic factors of the 172 acute stroke patients for three-month outcome are listed in Table 3. Although no patient died during the follow-up period, 133 had good outcomes and 39 had poor outcomes. Statistical analysis revealed that history of coronary artery diseases, NIHSS score on admission, pre-existing statin use, and platelet activation markers (CD62P and CD63) were significantly different between the good and poor outcome groups. Variables used in the stepwise logistic regression model included underlying age, gender, lipid profiles, coronary artery diseases, NIHSS score, pre-existing statin use, and CD62P and CD63 levels. After analysis, only NIHSS score (odds ratio (OR) = 1.10, 95% confidence interval (CI) = 1.03-1.17; *P *= 0.007), underlying coronary artery diseases (OR = 0.05, 95% CI = 0.01-0.38; *P *= 0.003), and pre-existing statin use (OR = 4.82, 95% CI = 1.22-19.03; *P *= 0.025) were independently associated with three-month outcome.

**Table 3:** Prognostic factors of patients with acute ischemic stroke

	Good outcome (*n *= 133)	Poor outcome (*n *= 39)	*P *value
Age (year)			
Sex (female) (%)	32.3	46.2	0.11
Hypertension (%)	77.4	84.6	0.33
Diabetes mellitus (%)	38.3	48.7	0.25
Hyperlipidemia (%)	50.4	38.5	0.19
Coronary artery disease (%)	1.5	12.8	0.002
Intra-cranial atherosclerosis (%)	23.3	30.8	0.34
Stroke sub-type			
Small vessel disease (%)	57.1	48.7	0.24
Large vessel disease (%)	39.1	51.3	
Statin therapy groups			
Pre-existing statin use (%)	29.3	10.3	0.016
Median NIHSS (IQR) scores on admission	3 (1-5)	4 (3-8)	0.009
Laboratory data on admission			
White blood cells (X10^3^/ml)	7.5 ± 0.2	7.6 ± 0.4	0.69
Red blood cells (X10^6^/ml)	4.6 ± 0.1	4.6 ± 0.1	0.73
Platelet counts (X10^4^/ml)	21.2 ± 0.6	19.5 ± 0.7	0.06
Total cholesterol (mg/dl)	183.0 ± 4.2	187.8 ± 8.5	0.61
LDL-cholesterol (mg/dl)	113.7 ± 3.7	114.3 ± 6.7	0.93
Triglyceride (mg/dl)	143.1 ± 7.8	147.2 ± 8.5	0.82
HbA1c (%)	7.0 ± 0.2	7.4 ± 0.4	0.33
Systolic BP (mmHg)	143.4 ± 1.8	140.6 ± 3.5	0.46
Diastolic BP (mmHg)	83.8 ± 1.1	82.8 ± 2.1	0.67
Platelet markers on admission			
CD62P (%)†	3.45 ± 0.38	5.39 ± 0.89	0.024
CD63 (%)†	1.41 ± 0.12	1.99 ± 0.31	0.035

Good outcome, three-month mRS (0 to 2); poor outcome, three-month modified Rankin Scale (3 to 6).

BP, blood pressure; HbA1c, hemoglobin A1c; IQR, inter-quartile range; LDL, low density lipoprotein; NIHSS, National Institutes of Health Stroke Scale;

† The data was expressed as primitive data, however, the *p *value was obtained from logarithmically transformed of primitive data to improved normality.

### Early neurologic deterioration and statin therapy

Of 172 acute ischemic patients, 14 suffered from END while hospitalized during the study period. Of the 14 END patients, four had hemorrhagic transformation, five had larger cerebral infarction, and five had increased of cerebral edema by follow-up brain CT. Patients with statin treatment after acute stroke (e.g. pre-existing statin use and statin-initiated groups) had significantly lower incidence of END (5 in 109 cases) compared with those without statin treatment (9 in 63 cases; *p *< 0.05). Furthermore, statin initiation after acute stroke also had significantly lower incidence of END (1 in 66 cases) compared with those without statin treatment (9 in 63 cases; *P *= 0.008).

## Discussion

The present study examined the expression of serial platelet activation markers (CD62P and CD63) and the three-month outcome after acute non-cardio-embolic ischemic stroke and produced four major findings. First, the severity of the initial stroke (NIHSS score on admission) is relatively low in patients with statin treatment before stroke onset. Second, CD62P and CD63 expressions in platelets on admission are significantly lower in patients with pre-existing statin use than those without pre-existing statin use. Furthermore, CD62P expressions between groups at four different time points (< 48 hours and on days 7, 30, and 90) are significantly different (*p *< 0.05). Third, underlying coronary artery diseases, NIHSS score on admission, and pre-existing statin use are independently associated with three-month outcome. Finally, patients with statin treatment in the acute phase of stroke have significantly lower incidences of END compared with those without statin treatment.

In the current study, platelet activation levels differed between the statin groups. However, they did not significantly predict good outcome. In contrast, statin use or non-use did predict outcome. This suggests that either platelet activity detected *in vitro *by flow cytometry may not actually reflect the *in vivo *platelet effects of statins, or that statins improve outcome via other mechanisms. The mechanisms by which statins benefit patients with acute ischemic stroke remain unclear and are likely to be multi-factorial. Increasing evidence shows that statins have pleiotropic effects beyond their lipid-lowering effects [25]. Statins interfere with platelet aggregation and have anti-inflammatory, anti-oxidative, and anti-apoptotic properties [26-29]. Previous studies have demonstrated that platelet aggregation and leukocyte activity are significantly increased after ischemic stroke and contribute to the severity of brain damage [4,30]. The present study is the first to demonstrate that platelet activation markers (CD62P and CD63) are significantly inhibited in patients taking statins prior to an acute non-cardio-embolic ischemic stroke. These results are consistent with those of previous studies and further corroborate the anti-platelet effect of statins [31]. This anti-platelet effect may be via improved endothelial function and increased systemic nitric oxide bioavailability that inhibits exaggerated platelet activation [26].

There is mounting evidence that statin therapy may alter vascular atherosclerosis and reduce cardiovascular events, including myocardial infarction, stroke, and death [13,14,32]. The JUPITER study further demonstrates that rosuvastatin significantly reduces the incidence of major cardiovascular events in apparently healthy persons without hyperlipidemia but with elevated high-sensitivity C-reactive protein levels [33]. Furthermore, the abrupt withdrawal of statin therapy may increase the risk of cardiovascular events and death because of rebound of inflammatory response [23,34,35].

Recent studies also show that prior or early use of statins may reduce the severity and improve the outcome of myocardial infarction, ischemic stroke, and intra-cerebral hemorrhage [17-20]. Consistent with previous research, the current study reveals that pre-treatment with statins may be associated with reduced clinical severity and favorable three-month outcome in patients with acute ischemic stroke. Recent clinical research demonstrates that high CD62P and CD40L expressions on admission are strongly associated with poor clinical outcomes after acute ischemic stroke [36]. Therefore, stronger inhibition of platelet activity in the acute phase of ischemic stroke may be beneficial in improving clinical outcome.

The causes of END after acute ischemic stroke remain unknown but may be related to hemorrhagic transformation, progression of cerebral infarction, or cerebral edema. Previous researchers hypothesize that early leukocyte-endothelium interaction and substantial inflammatory reaction may play important roles in patients with stroke in progression [4,19]. In the current study, although the causes of END are heterogeneous between groups, statin treatment in the acute phase of stroke reduces the incidence of END compared with those without statin treatment after stroke. Because of the limitations of case number and study design, the role of statin therapy in END cannot be explained.

This study has several limitations. First, this is an observational study and it is possible that unmeasured or unknown confounders may have influenced the results. Second, although patients with prior anti-platelet therapy before acute stroke have been excluded, the expression of the platelet activation markers may be influenced by other drugs (e.g. beta-blocker, ACE inhibitors, and calcium channel blockers) that may cause potential bias in the statistical analysis [37]. Third, whether any particular statin or dosage has superior effects has not been tested because of the relatively small number of patients. Fourth, the baseline NIHSS is lower in the pre-existing statin use group. This may be based on very early effects of statins (such as increased perfusion and tissue salvage/collateral flow, which would be plausible if NIHSS measurement was performed at 12 hours after stroke onset) or may represent an imbalance in stroke severity between the two groups suggesting a bias of study design because it is not randomized. Fifth, patients with cardio-embolic stroke were excluded from this study according to medical history, ECG, and/or echocardiography. Thus, the effect of statin pre-treatment in patients with cardio-embolic stroke was unclear. Finally, this is a cohort study in a single-center with a relatively small patient number. Thus, it is possible that there is a smaller-than-expected magnitude of the protective effects exerted by statins. Randomized, larger-scale trials are warranted to establish whether early statin therapy has important beneficial effects in patients with acute ischemic stroke.

## Conclusions

Statin therapy in acute non-cardio-embolic ischemic stroke can improve the three-month outcome and prevent END through potential anti-platelet effects. More prospective, longitudinal observational studies are warranted to evaluate the relation between dosage and choice of different statins in treating non-cardio-embolic stroke patients, to determine how to prevent END, and to improve neurologic outcome.

## Key messages

1. Prior statin therapy is associated with reduced platelet activity in patients with acute ischemic stroke.

2. Pre-treatment with statins is associated with the severity of acute ischemic stroke.

3. Our study confirms previous studies demonstrating that pre-existing statin use is associated with improved clinical outcomes in acute ischemic stroke.

## Abbreviations

ANOVA: analysis of variance; APTT: activated partial thromboplastin time; BI: Barthel index; CI: confidence interval; DM: diabetes mellitus; END: early neurologic deterioration; HbA1c: hemoglobin A1c; HDL: high density lipoprotein; HMG-CoA: 3-hydroxy 3-methyl-glutaryl coenzyme-A; LDL: low density lipoprotein; MRA: magnetic resonance angiography; MRI: magnetic resonance imaging; mRS: modified Rankin Scale; NIHSS: National Institutes of Health Stroke Scale; OR: odds ratio; PE: phycoerythrin; PT: prothrombin time; RBC: red blood cell; WBC: white blood cell.

## Competing interests

The authors declare that they have no competing interests.
